# Changes in Hematological Parameters and Liver Enzymes During Laparoscopic Cholecystectomy

**DOI:** 10.7759/cureus.13098

**Published:** 2021-02-03

**Authors:** Adam Umair Ashraf Butt, Ahsan Sajjad, Abdur Rehman Malik, Ahmad Farooq, Qasim Ali, Zuhair Ali Rizvi, Muhammad Sarfraz Khan, Muhammad Anwar

**Affiliations:** 1 Urology, Rawalpindi Medical University, Rawalpindi, PAK; 2 Medicine, Rawalpindi Medical University, Rawalpindi, PAK; 3 Neurology, Rawalpindi Medical University, Rawalpindi, PAK; 4 Medicine, Government Rural Dispensary, Rawalpindi, PAK; 5 General Surgery, Holy Family Hospital, Rawalpindi, PAK; 6 Surgery, Holy Family Hospital, Rawalpindi, PAK; 7 Intensive Care Unit, Shifa International Hospital, Islamabad, PAK; 8 General Surgery, Rawalpindi Medical University, Rawalpindi, PAK; 9 Surgery, Rawalpindi Medical University, Rawalpindi, PAK

**Keywords:** laparoscopic cholecystectomy, liver function tests, mean platelet volume, cholelithiasis, length of stay

## Abstract

Background

Changes in hematological parameters, such as neutrophils, leukocytes, neutrophil-lymphocyte ratio, platelet lymphocyte ratio, and mean platelet volume, have been observed during laparoscopic surgeries.

Objectives

The objectives of this research were to assess the changes in hematological parameters and liver enzymes during laparoscopic cholecystectomy (LC).

Methods

This prospective observational study included patients undergoing laparoscopic cholecystectomy for symptomatic cholelithiasis. Patients with comorbidities, including hepatitis, diabetes, and where laparoscopic cholecystectomy was converted to open cholecystectomy, were excluded. Preoperative and postoperative baseline hematological parameters and liver function tests (LFTs) were recorded. Characteristics like age, gender, body mass index (BMI), indication for surgery, duration of surgery, the pressure of pneumoperitoneum, and the duration of hospital stay were noted. A paired sample t-test was applied to assess the difference between the mean pre and postoperative values of different hematological parameters.

Results

It was observed that hemoglobin (Hb), hematocrit (Hct), platelets, and alkaline phosphatase (ALP) decreased postoperatively. However, mean corpuscular volume (MCV), mean platelet volume (MPV), leukocytes, and alanine transaminase (ALT) increased postoperatively. The difference in mean Hb, MCV, Hct, leukocytes, MPV, and ALT was statistically significant (p<0.05).

Conclusion

There were significant changes in the levels of hematological parameters and liver enzymes during LC.

## Introduction

Cholelithiasis is a prevalent disease throughout the world, contributing a prevalence of 10%-15% in the western population and 3%-5% in African and Asian populations [1]. In Pakistan, a study noted a prevalence of 10.2% for cholelithiasis among participants undergoing ultrasonography [2]. Laparoscopic cholecystectomy (LC), one of the most commonly performed procedures for gallbladder diseases, has replaced open cholecystectomy (OC) since the 1990s and is now considered a surgical procedure of choice for the removal of symptomatic gall bladder disease/symptomatic cholelithiasis [3].

Despite the short hospital stay and quick recovery period, laparoscopic cholecystectomy has a risk of developing cardiovascular complications in susceptible populations due to its hemodynamic and ventilatory consequences. The increased pressure of pneumoperitoneum and hypercarbia are associated with changes in hemodynamic parameters during laparoscopic surgeries [4-6]. The cost of pneumoperitoneal pressure peri-operatively is paid in the form of compressed abdominal vasculature, including inferior vena cava, aorta, splanchnic, hepatic artery, portal veins, and renal vasculature. Decreased hepatic artery and portal venous blood flow result in transient hepatocellular ischemia triggering cellular injury, as a consequence, the serum ALT level elevates. Higher intra-abdominal pressure is linked with higher fluctuations in hemodynamics, and more abnormalities in liver function tests (LFTs). Changes in LFTs postoperatively during laparoscopic cholecystectomy are related to pneumoperitoneum and duration of exposure [4,7-9].

Various studies have reported changes in hematological parameters during laparoscopic procedures [10-11]. Statistically, a significant difference was stated between pre and post insufflation values of leukocytes, neutrophils, neutrophil-lymphocyte ratio, platelet lymphocyte ratio, and mean platelet volume (MPV). A rise in MPV with increasing intraabdominal pressures has the clinical application of determining intra-abdominal hypertension [10-11]. A study reported the effects of CO_2_ insufflation on increased thromboembolic episodes due to increased coagulation factors and the diminished activity of the fibrinolytic system. This increase in thromboembolic events was directly associated with the pressure of the pneumoperitoneum and the duration of surgery [12].

The effects of these surgeries, under different circumstances, on the hematological parameters need to be clearly defined so that adequate measures are taken pre-, peri-, and postoperatively to minimize morbidity, mortality, hospitalization, as well as the overall cost for the patient and the hospital. In accordance with all these changes in various parameters reported by multiple studies, the primary purpose of conducting this study was to compare the preoperative and postoperative levels of hemoglobin (Hb), MCV, mean corpuscular haemoglobin (MCH), mean corpuscular haemoglobin concentration (MCHC), hematocrit (Hct), platelets, mean platelet volume (MPV), and LFTs like alanine transaminase (ALT) and alkaline phosphatase (ALP) in patients undergoing laparoscopic cholecystectomy.

## Materials and methods

This was a prospective observational study conducted from June to November 2019 at the surgical unit of a tertiary care hospital in a developing country. A total of 54 patients, out of which eight were male and 46 were female with a suspected diagnosis of cholelithiasis were identified. Confirmation was done through LFTs and abdominal ultrasounds. The inclusion criteria consisted of: those having symptomatic gall bladder disease, patients having American Society of Anaesthesiologists (ASA) physical status I or II, and those undergoing laparoscopic procedures. The patients suffering from any disease affecting MPV (such as diabetes and stroke), those in which LC was converted to OC, and those suffering from postoperative infections or coagulation disorders were excluded from this study.

Blood (2 cc) was drawn from the ante-cubital vein under aseptic conditions. These blood samples, drawn within 12 hours of the procedure, were used to determine the baseline serum ALT, ALP, leukocytes, MPV, Hb, MCV, MCH, MCHC, Hct, and platelets of the patients. The preoperative and postoperative values of these parameters were determined.

The Rotofix-32 (Hettich, Massachusetts) machine was used to centrifuge the blood for three to five minutes. The Beckman Coulter AU-480 automation machine (Brea, California) was used to check ALT levels. In preop rooms, the vitals of patients were maintained within normal limits and carefully monitored for 24 hours.

Under general anesthesia, standard four-port laparoscopic cholecystectomy was performed. Pneumoperitoneum was established with the closed technique with a veress needle. The intra-abdominal pressure used and the duration of surgery were recorded. Within 12 hours, 2 cc blood was again drawn from the ante-cubital vein following the same procedure to determine postoperation levels for the variables under study. Other variables like age, gender, BMI, and length of hospital stay were also noted.

A paired sample t-test was applied to test the difference between the mean pre and postoperative values of different hematological parameters and liver enzymes. An independent sample t-test was used to examine changes in the parameters between males and females. Finally, analysis of variance (ANOVA) was conducted to assess the changes in the parameters between different BMI groups. A two-tailed p < .05 was considered statistically significant. The analysis was carried out using the Statistical Package for Social Sciences (SPSS) v.23.0 (IBM Corp, Armonk, NY).

## Results

Demography

A total of 54 patients were selected for the study. Out of these, eight (14.8%) were male and 46 (85.2%) were female. The mean age of patients was 42.3519 (SD=15.69). The mean duration of the surgery was 51.02 min (SD=18.18) (range=20-90). While the mean pressure of the peritoneum was 13.54 mmHg (SD=1.89) (range=11-16). All the patients had symptomatic cholelithiasis. Fifty-two patients had a postoperative stay of less than 24 hours after their surgery while one stayed for two days and one for three days. Table 1 shows the gender-wise distribution of various age groups.

**Table 1:** Gender-wise distribution in various age groups

Age Group	Male (n=8)		Female (n=46)	
	(n)	(%)	(n)	(%)
20-39 yrs.	2	25	22	47.8
40-59 yrs.	3	37.5	19	41.3
60+ yrs.	3	37.5	5	10.9

Comparison of pre and postoperative hematological findings

A paired sample t-test was conducted to compare the levels of hematological factors between preop and postop. conditions. There was a significant rise in the ALT levels from preop (M=33.17, SD=39.69) to postop (M=55.61, SD=62.93) conditions; t (53) =-2.755, p =0.008. Similarly, MPV, MCV, and leukocytes also showed significant rises from preop to postop levels. On the other hand, there was a significant drop in the levels of hemoglobin from preop (M=12.833, SD=1.5694) to postop (M=12.1428, SD=1.6271) conditions; t (53) =4.119, p >0.001. Similarly, there was also a significant drop in hematocrit from preop (M=37.50, SD=4.34) to postop conditions (M=35.69, SD=3.93); t (53), p=0.001. Table 2 shows a detailed picture of the results of the test.

**Table 2:** Changes in hematological factors between preop and postop conditions 1) Hb=Haemoglobin, MCV=Mean Corpuscular Volume, MCH=Mean Corpuscular Haemoglobin, MCHC= Mean Corpuscular Haemoglobin Concentration, MPV=Mean Platelet Volume, ALT=Alanine Transaminase, ALP=Alkaline Phosphatase 2) *=significant value (p<0.05)

Hematological Factor	Preop		Postop		t	df	p-value
	Mean	S.D	Mean	S.D			
Hb (g/dL)	12.83	1.57	12.14	1.63	4.119	53	0.000*
MCV (fL)	80.49	8.69	82.51	6.79	-2.334	53	0.023*
MCH (pg)	27.82	3.05	28.10	3.35	-0.786	53	0.436
MCHC (g/dL)	33.65	2.20	33.82	2.75	-0.388	53	0.700
Hct	37.50	4.34	35.69	3.93	3.680	53	0.001*
Leukocytes (*10^3/IU)	8.43	2.44	12.54	3.73	-7.748	53	0.00*
Platelets (*10^3/uL)	303.28	79.34	286.07	80.54	1.901	53	0.063
MPV (fL)	8.92	0.87	9.21	0.69	-2.994	53	0.004*
ALT (U/L)	33.17	39.69	55.61	62.93	-2.755	53	0.008*
ALP (U/L)	152.78	120.06	143.79	117.24	0.819	53	0.416

Comparison of the change in levels between different groups

The difference between the preop and postop levels for all the variables was calculated. An independent sample t-test was applied to compare the changes between males and females. However, no significant difference between the genders could be found for any of the variables under the study. The ANOVA conducted to find the difference in the levels of the hematological factors during surgery, between the BMI groups, did not report any significant differences.

## Discussion

Laparoscopy has become one of the staples of modern surgery practice. Hence, its benefits and adverse effects need to be carefully monitored to continue to benefit society. We have, in our study, obtained some interesting results that will help provide a clearer picture as to the effects of this surgery. There were significant differences in the levels of some of the parameters under study. This suggests that despite its apparent innocuousness, laparoscopy still requires appropriate care and precautionary measures in order to assure the well-being of the patient.

The mean age of the participants in our study was 42 of which an overwhelming majority was female (85.2%). Several studies measuring the prevalence of the disease or relating to surgical procedures for cholelithiasis have reported similar figures [13]. A study in Taiwan also found a higher incidence of cholelithiasis in females [14]. This is expected, as several studies have shown the female gender to be a risk factor for the development of gallstones [15].

This study's primary purpose was to describe the changes that occur in the blood due to laparoscopic cholecystectomy. First of all, we found there was a significant drop in the Hb and hematocrit levels. These results were corroborated by Lindberg et al. [16]. This is easily explained by the dilutional effect of intravenous (I/V) fluids administered during the procedure. Moreover, the rise in the MCV is understandable as well. I/V fluids are administered throughout the procedure to avoid shock and postoperatively to prevent dehydration. Continued administration with minimal bleeding due to laparoscopic procedures leads to blood dilution and a drop in plasma osmolality. This, in turn, leads to a shift of water into cells, leading to a possible rise in MCV. Another variable to show rising levels postoperatively were leukocytes. This is supported by the study conducted by Bitkin et al. in Turkey [11]. This is because the trauma caused during surgery will activate immune pathways, which will inevitably lead to a rise in leukocyte counts.

The MPV is an essential marker for increased intra-abdominal pressure [11]. Thus, it makes sense that our results also showed a significant increase in MPV after the procedure, similar to the results reported by Bitkin et al. [11]. This rise is further corroborated by a study conducted by Celep et al. in Turkey [10].

However, a study by Marakis et al. found decreased mean platelet volume in their patients [17]. This is attributable to the increased thrombolytic activity, and coagulation activation found in their patients and is reported by different studies [16,18]. This leads to the platelets being depleted, reflecting the fact that Marakis et al. also found a significant decrease in platelet count. In contrast, there was no significant difference in platelet count in our study [17].

In support of our results, a study by Crema et al. showed no significant difference in platelet counts [19]. Our results, which show no significant change in platelet counts but an increase in mean platelet volume, are explained by studies that report that in stressful situations, such as surgical procedures, initially, only increased volume platelets are mobilized. An increase in the absolute number of circulating cells occurs after at least one to two days [20-21].

The final variable under study that showed a significant rise during the procedure was ALT. Various studies show similarly significant results [7,22-23]. These changes are transient, and the enzyme levels return to normal after three to 10 days [24]. The normal intra-abdominal pressure is 8 mmHg. However, during laparoscopy, the pneumoperitoneum created exerts an increased pressure of about 13-14 mmHg. Pneumoperitoneum-induced hepatic hypoperfusion has been demonstrated in different experimental and clinical studies, such as one by Jakimowicz et al., which showed that laparoscopic insufflation reduces portal venous flow [25]. This, in turn, leads to hepatic injury, leading to the rise in ALT levels. ALP levels, on the other hand, did not change significantly, similar to results obtained in different studies such as the one done by Hasukic et al. [23].

There are a few limitations in this study. First, data were collected from a single institution. Furthermore, the male to female ratio was very unbalanced so a proper comparison between the two genders could not be established. Furthermore, this being a prospective observational study, it was very time-consuming, which resulted in the sample size being small. Thus, a study with a larger sample size should be performed to obtain results that are better generalizable.

## Conclusions

There were significant changes in the levels of hematological parameters and liver enzymes. Care must be taken to avoid deranging these factors to dangerous levels so that further complications and morbidity can be avoided. Furthermore, this study can serve as a springboard for people to investigate further how these parameters change in light of various controllable factors during the procedure and what we can do to minimize these changes.
